# Comparing Implementation and Effectiveness Outcomes for Two Implementation Strategies of the Keep It Up! Digital HIV Prevention Program: A Type 3 Hybrid Effectiveness-Implementation Trial

**DOI:** 10.1007/s10461-025-04838-0

**Published:** 2025-08-19

**Authors:** Brian Mustanski, Nanette Benbow, Kathryn Macapagal, Dennis Li, Krystal Madkins, Rana Saber, Benjamin Linas, JD Smith, C. Hendricks Brown, Sarah Munroe, Susheel Reddy, Bruce R. Schackman, Gregory Swann, Patrick Janulis, Alithia zamantakis, Juan Pablo Zapata

**Affiliations:** 1 Impact Institute, Northwestern University, 625 N. Michigan Ave. Floor 14, Chicago, IL 60611 USA; 2 Department of Medical Social Sciences, Feinberg School of Medicine, Northwestern University, Chicago, IL USA; 3 Department of Psychiatry and Behavioral Sciences, Feinberg School of Medicine, Northwestern University, Chicago, IL USA; 4 Galter Health Sciences Library and Learning Center, Feinberg School of Medicine at Northwestern University, Chicago, IL USA; 5 Section of Infectious Disease, Boston Medical Center, Boston, MA USA; 6 Department of Population Health Sciences, School of Medicine, The University of Utah, Salt Lake City, UT USA; 7 Division of Infectious Diseases, Department of Medicine, Feinberg School of Medicine, Northwestern University, Chicago, IL USA; 8 Department of Population Health Sciences, Weill Cornell Medical College, Cornell University, New York, NY USA

**Keywords:** HIV/AIDS, Men who have sex with men, Digital health interventions, Type III hybrid effectiveness implementation trial

## Abstract

This study sought to compare implementation and effectiveness outcomes for two delivery strategies of a digital HIV prevention intervention: community-based organization (CBO) versus direct-to-consumer (DTC). A type III hybrid effectiveness–implementation trial compared two implementation strategies of *Keep It Up*! (KIU! ) for young men who have sex with men (YMSM) (*n* = 2124). Data were collected at baseline and 12-weeks in 2019–2023. DTC enrolled more participants, but CBO recruited more Black (11.7% vs. 21.7%) and Latino YMSM (27.3% vs. 32.9%). CBO enrollees were at higher HIV risk based on condomless anal sex (*p* < .05) and rectal Gonorrhea rates (*p* < .01). Although pre-exposure prophylaxis (PrEP) use with adherence was 1.94 times higher at follow-up vs. baseline (*p* < .0001) across arms, PrEP increase was 1.60 times higher in CBO vs. DTC (*p* < .0001). CBO delivery averted more HIV infections per 100 people-years than DTC (1.65 vs. 0.62), but the cost of averting an HIV infection was higher in CBO than DTC ($983,029 vs. $173,313). Although CBOs were able to reach participants at higher HIV risk, the DTC strategy was estimated to cost less per infection averted and below established thresholds for cost savings. Findings must be interpreted within the context of data collection during the COVID-19 epidemic. More information about KIU! can be found at https://kiu.northwestern.edu/.

Trial registration: NCT03896776 (date of registration: 04/01/2019).

**Supplementary Information:**

The online version contains supplementary material available at 10.1007/s10461-025-04838-0.

Young gay, bisexual, and other men who have sex with men (YMSM) are disproportionally affected by HIV in the United States (U.S.). With an estimated 14,300 new infections in 2022, they represent 75% of infections among 13–34 year olds and 45% of total infections [1]. However, YMSM-focused HIV prevention interventions only represent 4% (5/117) of the risk reduction interventions included in the Centers for Disease Control and Prevention (CDC) Compendium of Evidence-Based Interventions and Best Practices for HIV Prevention [2], which is a scientific inequity driving a health disparity [3]. As prevention resources have shifted away from traditional face-to-face behavioral risk reduction interventions towards biomedical approaches such as pre-exposure prophylaxis (PrEP) and HIV treatment as prevention, scalable and low-cost “adjunctive interventions” (i.e., behavioral interventions that increase motivation, adherence, or engagement with an intervention) [4] delivered using public health approaches are needed to promote utilization among YMSM—this cannot be accomplished with equity purely through clinical care [5].

Digital health interventions (DHIs) may offer a solution given their low incremental costs of delivery and evidence of effectiveness on increasing adoption and sustainment of HIV testing, prevention (i.e., condoms, PrEP), and care [6, 7], but the implementation strategies necessary to scale up DHIs for widespread delivery in real-world settings have received little to no research [8]. The National Institutes of Health and other funders have invested heavily in the development and testing of HIV DHIs, producing many evidence-based programs [9–11], which makes the gap in understanding how to implement them particularly acute [8]. Effective implementation strategies for HIV DHIs can also serve as templates for other diseases.

*Keep It Up!* (KIU! ) is an HIV prevention DHI designed for diverse YMSM that uses interactive multimedia to increase sexual health knowledge, motivate safer behaviors, and encourage healthy relationships. Briefly, KIU! has been tested in multiple studies since 2009 [12–14] and, as the first DHI classified by the CDC as a “best evidence” HIV risk reduction intervention [14], is ideal for evaluating pragmatic, real-world implementation. Here, we report the results of a large, type III hybrid effectiveness–implementation trial [15] comparing Direct-to-Consumer (DTC) against Community-Based Organization (CBO)-mediated delivery. Both are viable strategies for disseminating DHIs. The DTC model is a widely used technology dissemination approach and is feasible to implement through a national technology coordinating center [8, 16, 17]. The CBO model is the current backbone of implementing HIV prevention programs. The effectiveness and relative advantages of these two delivery strategies are unknown, however, and little is known about their pragmatic implementation. The goals of this primary outcomes paper are: (1) to compare implementation characteristics, reach to highest risk populations, and delivery costs of the two strategies, and (2) to estimate the effectiveness on changing key HIV prevention outcomes and cost per infection averted by delivery strategy.

## Methods

### Participants and Procedures

Detailed information on the hybrid trial design is available in a protocol paper, including specification of design modifications adopted during the COVID-19 pandemic, sample size targets, and process and economic evaluation [18]. In brief, 66 U.S. counties with high numbers of YMSM were identified and randomized two-to-one (CBO: DTC) to delivery strategy. Forty-four counties were eligible for CBOs to apply based on an estimated population of YMSM surpassing 1500 individuals (as estimated using a model of men who have sex with men MSM population size [19]), no previous KIU! studies, and minimizing bordering counties to mitigate potential inadvertent exposure across jurisdictional lines. A request for proposals was issued in these 44 counties for CBOs to receive funding to integrate KIU! into routine HIV testing of YMSM and to participate in limited research activities. Reviews of applications were conducted using a pragmatic study section approach led by an experienced public health agency leader that mimicked standard practice. As planned, 22 CBOs in distinct and noncontiguous counties were selected and funded to implement KIU!. In the initial design, the DTC strategy was to only recruit participants in the 22 counties randomized to this strategy, but due to a variety of pragmatic considerations (e.g., COVID-19 restrictions, changes to social media geotargeting capabilities), we decided to switch to a national recruitment strategy (excluding participants living in the 22 CBO counties). This strategy is reflective of the delivery approach used in many previous studies of DHI delivery.

YMSM research eligibility criteria included: aged 18–34, proficient in English, assigned male sex at birth, identified as either a man or gender non-conforming, tested negative for HIV or self-reported a recent negative test, reported sex with or attraction to men, reported either not using HIV PrEP or missing a PrEP dose in the previous six months, and completed the baseline survey. Data collection occurred between December 2019 to March 2023. The protocol was approved by the Northwestern University Institutional Review Board.

### Intervention Delivery

KIU! is a custom mobile- and computer-responsive web-based participant-facing application plus an online administrative dashboard with user roles of varying levels of access. KIU! was developed to allow for the creation of multiple and customizable instances of the intervention that can be accessed via a single login page. The content and data associated with each instance of KIU! was only accessible to the participants and implementers assigned to a specific CBO or DTC instance. Customizable features of the KIU! application included tailored introductory videos and content pages, resource lists, automated email reminder content, and the inclusion of service request forms that connected participants to services offered by the CBO or nationally. The administrative dashboard allowed implementers to register participants to their instance of KIU! and view real-time data about their participants, including a participant tracker to track progress through KIU! and a to-do list feature to identify participants that needed follow-up. Online training was developed for implementers covering how to use the administrative dashboard, YMSM recruitment strategies, and retention best-practices. Ongoing technical assistance and training was provided to CBOs, most of which were delivering a DHI for the first time. The DTC arm acted like a standalone CBO but was implemented in-house by trained research staff with experience working with YMSM. For more information related to the content, delivery and customization of the intervention see study protocol [18].

### Data Collection

Participants provided informed consent and completed a baseline survey prior to accessing the intervention content via the KIU! application. Participants received booster content at 6 and 12 weeks after baseline. At 12 weeks, participants also completed a post-intervention survey before viewing booster content.

### Reach and Effectiveness Outcomes

Our primary outcome was a model-based estimate of HIV infections averted, which incorporated aspects of both reach (number of participants recruited, baseline Rectal Gonorrhea (RG) test results, baseline PrEP) and effectiveness (change in RG rates and PrEP at follow-up) to quantify prevented HIV infections. As such, it is one operationalization of the concept of “public health impact,” which is defined as Reach times Effectiveness [20]. RG test results were of particular interest because of the strong correlation found between RG and HIV incidence among MSM not on PrEP [21]. Secondary effectiveness outcomes included change from baseline to follow-up in RG rates, number of male condomless anal sex (CAS) partners, and adherent PrEP use (aPrEP). Implementation outcomes included number of participants enrolled, intervention engagement, and the HIV risk profile of participants based on: demographics (particularly Black and Latino participants given highest HIV incidence [22, 23]), HIV risk behaviors, and aPrEP—as reaching participants at elevated HIV risk is an important component of an effective implementation strategy. Outcomes were pre-specified [18], and the trial was registered.

### HIV Infections Averted

HIV infections averted were calculated as the difference between baseline and 12 week extrapolated HIV incidence rates (IR) per 100 person-years. Given the incidence of HIV among YMSM in the U.S [22, 23]., very large sample sizes would be required to directly estimate HIV infections averted. Accordingly, HIV incidence rates were estimated for each study-arm timepoint from RG infections using a predictive model of HIV incidence published by Mullick and Murray [21]. See Online Resource Supplemental Methods for details. Because aPrEP has no effect on RG incidence but does prevent HIV infection, PrEP adherence would bias the Mulick and Murray method if no correction was made for the dissociation of RG from HIV during aPrEP use. To approximate the effect of aPrEP on regression-predicted HIV IRs, least square (LS) mean estimates from the Mulick and Murray regression using the quantity (RG IRs multiplied by the projected proportion of person-time not adherent to PrEP) were used to obtain aPrEP-adjusted, extrapolated HIV IRs for each of the four study-arm timepoints. See the descriptions provided as part of Table 3 for full details on both this adjustment for PrEP use and additional details on the calculations of HIV infections averted.

### Self-Report Measures

Race and ethnicity, age, gender, and sexual orientation were collected at baseline. Study participants reported the number of men they had CAS with in the previous 3 months as part of the baseline and follow-up surveys. Current PrEP use was assessed with the item: “Are you CURRENTLY taking PrEP to reduce the risk of getting HIV?” with “yes” and “no” response options. Current PrEP users were asked if they took PrEP every day or on demand (i.e., “event driven” or “2-1-1”). Daily PrEP users were asked, “In the last MONTH (30 days), has there been a time when you did NOT take PrEP for 4 or more days in a row?”, and PrEP users who reported taking PrEP on demand were asked, “You indicated using PrEP “on demand” or with a “2-1-1” dosing schedule. In the last MONTH (30 days), has there been a time when you MISSED any doses of PrEP before or after having sex?” Participants who reported that they did not miss doses as described above were coded as aPrEP (1), and participants who reported either missing doses or not using PrEP were coded as aPrEP non-users (0).

### Implementation Costs

We employed a micro-costing approach to assess costs associated with implementing KIU! in the CBO and DTC arms [24]. To ensure comprehensive enumeration of all resources consumed, we categorized costs as (1) start-up, or one-time costs necessary for program launch; (2) variable, or costs that increase as a function of the number of participants enrolled; and (3) time-dependent, or costs that increase as a function of program duration. We used standardized expense reports to collect all resource consumption in the DTC arm and resources consumed centrally in the CBO arm. We conducted interviews at all 22 participating CBO sites to assess the resources consumed at the decentralized site level. We estimated total resource consumption in the CBO arm by summing the resources consumed centrally and the resources consumed at all 22 CBO sites.

### Statistical Analyses

Differences in distribution of categorical variables by study arm were evaluated using chi-square tests for nominal categorical variables and the chi-square correlation statistic for ordinal categorical variables. Exact methodologies were applied in instances of sparse cell sizes. For continuous variables, differences were evaluated using two-sample t-tests assuming unequal variances employing Satterthwaite degrees of freedom.

Multivariable analyses evaluating the outcomes of RG incidence, number of male CAS partners, and aPrEP were applied using repeated measures generalized estimating equation (GEE) models with study arm, timepoint, and study-arm-by-timepoint interaction terms as categorical independent variables. Use of the most parsimonious models allowed for estimates of effectiveness that are essentially unadjusted—reflecting the real-world differences introduced by nascent sociodemographic differences between participants enrolled using the respective engagement strategies. Marginal models in these instances were favored as they produced “population-averaged” estimates of effect that did not require explicit declaration of the correlation structure inherent to these data. Correlations between observations from the same subject within CBO occurred as a function of the de facto paired design and were accommodated using an unstructured correlation matrix. The DTC arm was treated as a single, distinct site when considering correlative effects. A Poisson probability distribution was specified for the models with RG incidence and aPrEP as the outcome; a normal distribution was specified for the model with number of male CAS partners as the outcome. LS mean estimates or rate ratios were used to evaluate differences by study arm and/or timepoint.

#### Costing Data Analysis

After quantifying all resources, we multiplied the resource utilization estimates collected through the expense reports and site interviews by standardized unit costs to estimate total costs. We applied distinct literature-based overhead ratios of indirect costs to labor costs on the centralized and decentralized site level [25]. Efficiency was determined by assessing the cost per infection averted, excluding startup costs because those costs will not reincur in future implementation. A comprehensive micro-costing analysis accounting falls outside the scope of this primary outcomes paper; however, a detailed report of costs is published [26].

#### Multiple Imputation Approach to Missing Data

Results were missing for more than 50% of the observations for all tested infections at all time points (RG baseline: 55.7%, 12 week: 58.8%). Survey responses relevant to aPrEP and sexual behavior were missing for 43.2% of the observations at follow-up. Fully conditional specification multiple imputation was used to accommodate missingness. An imputation model containing the following covariates was fit: RG test result, study arm, timepoint, race, categorical age, sexual orientation, aPrEP, PrEP use, number of male anal sex partners with no condom, number of anal/vaginal sex partners in past 3 months, number of completed intervention modules, rectal chlamydia test result, urethral gonorrhea test result, urethral chlamydia test result, and a study arm by timepoint interaction term. An indicator variable for the interaction term was used in the imputation model and then exchanged in all subsequent analysis models with the product of the main effects. Terms in the imputation model were selected if they were needed in the analysis models, demonstrated evidence of association with the effectiveness outcomes, or demonstrated evidence of association with the missingness of the effectiveness outcomes. Sixty imputations were performed. Loss to follow-up by recruitment strategy, demographic characteristics, auxiliary variables used in the imputation model, and outcomes are described in Online Resource Table S1. Differences by recruitment strategy for all auxiliary variables used in the imputation model are reported in Online Resource Table S2.

#### Sensitivity Analyses for HIV Infections Averted

Sensitivity analyses focused on variations on the assumed person-time on PrEP based on 30-day adherence data. As described above, our primary outcome model was conservative and assumed that for those who initiated PrEP between visits, it occurred halfway between visits (i.e., 1.5 person-months of coverage). A second analysis presented estimates of HIV infections averted with an augmented adjustment for PrEP that assumed PrEP initiation occurred right after intervention completion (i.e., 3 person-months of coverage). In order to help understand how our application of the Mullick and Murray [21] equation would estimate infections averted without the added correction for aPrEP use, an additional sensitivity analysis estimated HIV infections averted with no aPrEP adjustment (i.e., aPrEP use is ineffective at reducing HIV risk). Full details on these variations can be found in the description accompanying Table 3.

An additional component introducing a degree of uncertainty onto our estimates is the use of the Mulick and Murray formula itself. The confidence intervals associated with the HIV infections averted estimates were provided as a means of acknowledging the use of RG infection as a predictor for HIV infection. Both the application of varying assumptions regarding the effects of person-time on PrEP and the use of the confidence intervals addressing the uncertainty associated with the Mulick and Murray-derived estimates seek to establish a range of plausible values of HIV infections averted that can be ascribed to each treatment arm.

## Results

### Demographics

**Fig. 1:**
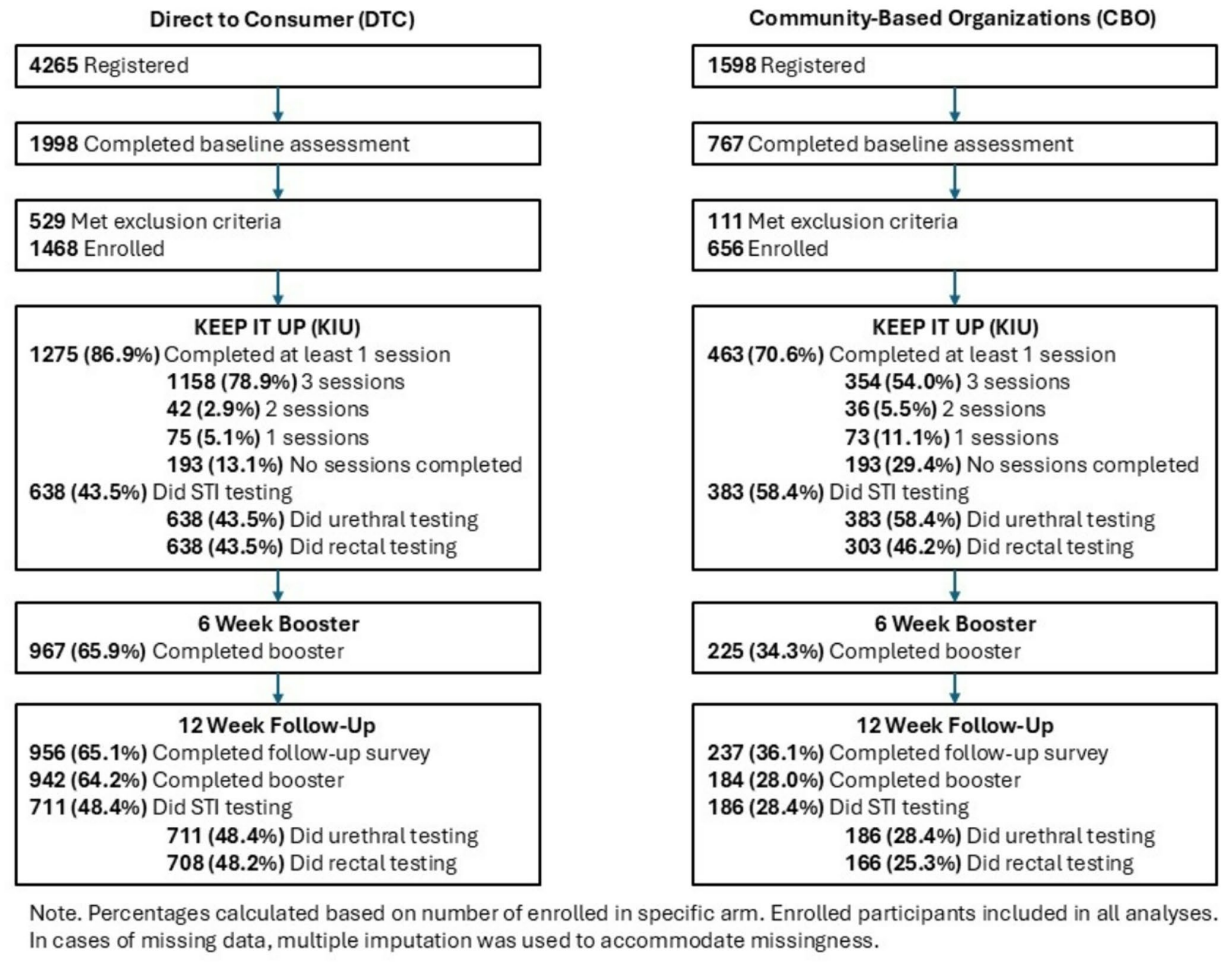
Participant Flow in the Keep It Up! Type III Hybrid Effectiveness–Implementation Trial, 2020–2023

The number of enrolled participants across both arms was 2,124 (see Fig. 1 for Participant Flow CONSORT map). Among enrolled participants, 55.7% received a negative test for HIV, and 44.3% self-reported a negative status at baseline. Demographic characteristics are available in Table 1.

**Table 1:** Demographics and outcomes of keep it up participants by recruitment strategy: 2020–2023

	DTC (*n* = 1468)	CBO (*n* = 656)	*P*-value	Total (*n* = 2124)
Age at baseline (years), n (%)			0.0508^a^	
18–21	331 (22.6)	110 (16.8)		441 (20.8)
22–25	418 (28.5)	198 (30.2)		616 (29.0)
26–29	400 (27.3)	207 (31.6)		607 (28.6)
> 30	319 (21.7)	141 (21.5)		460 (21.7)
Gender at baseline, n (%)			0.3918^b^	
Man	1369 (93.3)	605 (92.2)		1974 (92.9)
Gender nonconforming	99 (6.7)	51 (7.8)		150 (7.1)
Sexual brientation at baseline, n (%)			0.0015^b^	
Gay	1049 (71.5)	512 (78.1)		1561 (73.5)
Other	419 (28.5)	144 (22.0)		563 (26.5)
Race/ethnicity at baseline, n (%)			< 0.0001^b^	
White	622 (42.4)	208 (31.7)		830 (39.1)
Black or African-American	171 (11.7)	142 (21.7)		313 (14.7)
Latinx	401 (27.3)	216 (32.9)		617 (29.1)
Other	274 (18.7)	90 (13.7)		364 (17.1)
aPrEP last 30 days – baseline, n (%)			0.0893^a^	
Yes	242 (16.5)	128 (19.5)		370 (17.4)
No	1226 (83.5)	528 (80.5)		1754 (82.6)
Missing^c^	0	0		0
aPrEP last 30 days – follow-up, n (%)			< 0.0001^a^	
Yes	265 (18.1)	101 (15.4)		366 (17.2)
No	700 (47.7)	140 (21.3)		840 (39.6)
Missing^c^	503 (34.3)	415 (63.3)		918 (43.2)
Rectal Gonorrhea lab result – baseline, n (%)			0.0002^b^	
Positive	13 (0.9)	21 (3.2)		34 (1.6)
Negative	626 (42.6)	282 (43.0)		908 (42.8)
Missing, not done, or Invalid^c^	829 (56.5)	353 (53.8)		1182 (55.7)
Rectal Gonorrhea lab result – follow-up, n (%)			0.0015^d^	
Positive	10 (0.7)	10 (1.5)		20 (0.9)
Negative	699 (47.6)	156 (23.8)		855 (40.3)
Missing, not done, or Invalid^c^	759 (51.7)	490 (74.7)		1249 (58.8)
Number of Cisgender male sex partners in past 3 months, no condom – baseline, n (%)			0.6883^e^	
n	1468	656		2124
Mean (SD)	2.2 (4.32)	2.3 (4.30)		2.2 (4.31)
Median	1.0	1.0		1.0
IQR	0–2.0	0–3.0		0–3.0
Minimum, maximum	0, 70	0, 80		0, 80
Number of Cisgender male sex partners in past 3 months, No condom – follow-up, n (%)			0.5523^e^	
n	966	241		1207
Mean (SD)	1.7 (2.87)	1.8 (3.58)		1.7 (3.02)
Median	1.0	1.0		1.0
IQR	0–2.0	0–2.0		0–2.0
Minimum, maximum	0, 25	0, 35		0, 35
Number of intervention modules completed			< 0.0001^e^	
n	1468	656		2124
Mean (SD)	7.2 (3.08)	5.1 (3.64)		6.5 (3.40)
Median	9.0	7.0		9.0
IQR	7.0–9.0	0–9.0		4.0–9.0
Minimum, maximum	0, 9	0, 9		0, 9

Note: aPrEP = adherent PrEP use

^a^P-value calculated using the chi-square correlation statistic.

^b^P-value calculated using a chi-square test.

^c^Not considered when calculating p-value.

^d^P-value calculated using a Fisher’s exact test.

^e^P-value calculated using a two-sample t-test assuming unequal variances using Satterthwaite degrees of freedom.

### Reach Outcomes

Study arm differences for demographic characteristics, auxiliary variables, and outcomes are presented in Table 1. The DTC arm enrolled over twice as many participants as the CBO arm. The racial/ethnic composition of the two study arms was significantly different (*p* <.0001); the DTC arm recruited a higher proportion of White (42.4% vs. 31.7%), Asian/Pacific Islander (11.1% vs. 8.1%), and multiracial participants (6.7% vs. 4.1%) and a lower proportion of Black/African-American (11.7% vs. 21.7%) and Latino participants (27.3% vs. 32.9%) compared to the CBO arm.

Differences in rates of 30-day aPrEP by study arm at baseline were equivocal (CBO 19.5% vs. DTC 16.5%, *p* =.09). No difference was found in number of male CAS partners (CBO mean 2.3 vs. DTC mean 2.2, *p* =.69) at baseline. RG data at baseline were missing for a large percentage of the cohort. Analyses using imputed data that address missingness are presented in Online Resource Table S3. Using this imputed set, higher rates of RG were found for participants in the CBO arm at baseline (CBO 5.6%, vs. DTC 2.7% *p* <.001). Participants in the DTC arm were more engaged in the intervention as measured by number of modules completed (*p* <.0001).

### Effectiveness Outcomes

Multivariable analyses evaluating time and study arm trends simultaneously are shown in Table 2. RG rates among CBO participants were 2.07 times higher than those found in the DTC arm (CBO 4.8% vs. DTC 2.3%, *p* <.01), and the difference did not vary by timepoint. Although LS mean estimates of RG positivity at follow-up were lower in magnitude than those at baseline, these differences were not statistically significant.

**Table 2:** Multivariable effects of time and recruitment strategy on effectiveness outcomes using imputed Data^a^: 2020–2023

	RG positive test		Number of condomless Cisgender male sex partners		aPrEP	
	Least square mean estimate (95% CI)	*P*-value	Least square mean estimate (95% CI)	*P*-value	Least square mean estimate (95% CI)	*P*-value
Study arm						
CBO arm	0.048 (0.034, 0.068)		2.67 (2.22, 3.12)		0.293 (0.262, 0.329)	
DTC	0.023 (0.016, 0.034)		2.14 (1.94, 3.24)		0.213 (0.195, 0.233)	
CBO Arm: DTC Arm Rate Ratio/LS Mean Difference, (CBO – DTC)	2.07 (1.33, 3.23)	0.0013	0.53 (0.06, 1.00)	0.0272	1.38 (1.19, 1.59)	< 0.0001
Timepoint						
Baseline	0.033 (0.025, 0.044)		2.26 (2.07, 2.46)		0.179 (0.163, 0.198)	
Follow-up	0.025 (0.016, 0.042)		2.54 (2.11, 2.98)		0.349 (0.317, 0.384)	
Follow-up: Baseline Rate Ratio/ LS Mean Difference (FU – BL)	0.76 (0.46, 1.27)	0.2983	0.28 (−0.16, 0.72)	0.2069	1.94 (1.72, 2.19)	< 0.0001
CBO Arm						
Baseline	0.056 (0.040, 0.078)		2.35 (2.02, 2.68)		0.195 (0.167, 0.228)	
Follow-up	0.042 (0.025, 0.071)		2.99 (2.21, 3.76)		0.441 (0.380, 0.512)	
Follow-up: Baseline Rate Ratio/ LS Mean Difference (FU – BL)	0.75 (0.43, 1.30)	0.3031	0.64 (− 0.14, 1.42)	0.1067	2.26 (1.85, 2.76)	< 0.0001
DTC Arm						
Baseline	0.026 (0.018, 0.038)		2.17 (1.96, 2.38)		0.165 (0.147, 0.185)	
Follow-up	0.020 (0.011, 0.037)		2.10 (1.82, 2.38)		0.275 (0.248, 0.306)	
Follow-up: Baseline Rate Ratio/ LS Mean Difference (FU – BL)	0.77 (0.41, 1.44)	0.4190	− 0.08 (− 0.37, 0.22)	0.6165	1.67 (1.48, 1.89)	< 0.0001
Baseline						
CBO Arm: DTC Arm Rate Ratio/ LS Mean Difference (CBO – DTC)	2.11 (1.30, 3.42)	0.0024	0.17 (− 0.22, 0.56)	0.3838	1.18 (0.98, 1.44)	0.0876
Follow-up						
CBO Arm: DTC Arm Rate Ratio/ LS Mean Difference (CBO – DTC)	2.04 (1.07, 3.89)	0.0312	0.89 (0.11, 1.66)	0.0246	1.60 (1.35, 1.90)	< 0.0001

Note: *aPrEP *adherent PrEP use

^a^Least square mean estimates obtained using a repeated measures GEE model with study arm, timepoint, and a study arm by timepoint interaction term included as categorical independent variables. Correlation between observations from the same subject within CBO are accommodated through use of an unstructured covariance matrix. A Poisson probability distribution was specified for the models with rectal gonorrhea incidence and PrEP adherence as the outcome; a normal distribution was specified for the model with number of condomless cisgender male sex partners as the outcome.

Participants in the CBO arm reported significantly more male CAS partners when compared to DTC at follow-up (LS Mean difference in number of partners = 0.53, 95% CI: 0.06–1.00, *p* <.05). The difference between study arms was significant at follow-up (LS Mean = 0.89, 95% CI: 0.11–1.66, *p* <.05) but not at baseline (LS Mean = 0.17, 95% CI: −0.22–0.56, *p* =.38). The change in CAS partners from baseline to follow-up was not significant.

As shown in Table 2, differences in rates of aPrEP by study arm were equivocal at baseline (CBO 19.5% vs. DTC 16.5%, OR = 1.18, *p* =.09) and statistically significant at follow-up, with a 1.60 higher aPrEP rate at follow-up for the CBO arm (CBO 44.1% vs. DTC 27.5%, *p* <.0001). Increased aPrEP at follow-up was seen within both study arms, with a 126% (95% CI: 85–176%) increase in the CBO arm and a lower, but still significant, 67% (95% CI: 48–89%) increase in the DTC arm.

### HIV Infection Averted and Cost Per HIV Infection Averted

Extrapolated estimates of HIV incidence rates and infections averted are presented in Table 3. Our primary outcome was the estimate of HIV infections averted adjusted for the proportion of person-time on PrEP. CBO arm participants had a PrEP-adjusted HIV infections averted estimate of 1.65 infections/100 person-years (95% CI: 1.29–2.02), whereas the estimate of 0.62 infections/100 person-years (95% CI: 0.49–0.74) was significantly lower in the DTC arm. In comparison to the PrEP-adjusted estimates, participants across arms presented more conservative reductions in HIV incidence rates when evaluated via the sensitivity analysis without adjustment for aPrEP. Augmented adjustments for the proportion of person-time on PrEP (i.e., assuming if PrEP initiation occurred it happened right after intervention) were applied in a second sensitivity analysis. This analysis produced amplified adjustments in estimates of HIV infections averted of 0.98 (05% CI: 0.83–1.13) in the DTC arm and 2.77 (95% CI: 2.22–3.16) in the CBO arm, representing approximately 63% more infections averted per 100 person-years than our more conservative primary outcome estimate.

For our primary estimate of infections averted, the cost per HIV infections averted was $173,313 in the DTC arm and $983,029 in the CBO arm in 2021 dollars. Participants did not report any unintended effects or harms throughout the duration of this study.

**Table 3:** Estimates of HIV infections averted by recruitment strategy: 2020–2023

	DTC		CBO	
	Baseline	12 week follow-up	Baseline	12 week follow-up
Estimated RG positive test rate^a^ (95% CI)	0.027 (0.017, 0.036)	0.021 (0.008, 0.034)	0.056 (0.037, 0.075)	0.042 (0.020, 0.065)
RG IR^b^ - cases/100 py (95% CI)	10.63 (6.68, 14.57)	8.40 (3.28, 13.53)	22.36 (14.85, 29.87)	16.94 (7.93, 25.95)
No aPrEP adjustment (sensitivity analysis)				
Extrapolated HIV IR^c^ - cases/100 py (95% CI)	4.17 (3.23, 5.11)	3.68 (2.67, 4.69)	6.77 (5.73, 7.82)	5.57 (4.68, 6.46)
HIV infections averted per 100 py^d^ (95% CI)	**0.49** **(0.32**,** 0.66)**		**1.20** **(0.79**,** 1.62)**	
aPrEP adjusted (primary outcome)				
Proportion of person-time on PrEP	0.165^e^	0.220^f^	0.195^e^	0.319^f^
Proportion of person-time not on PrEP	0.835^e^	0.780^f^	0.805^e^	0.682^f^
Extrapolated HIV IR^g^ - cases/100 py (95% CI)	3.49 (2.70, 4.27)	2.87 (2.08, 3.66)	5.45 (4.61, 6.29)	3.80 (3.19, 4.41)
HIV infections averted per 100 py^d^ (95% CI)	**0.62** **(0.49**,** 0.74)**		**1.65** **(1.29**,** 2.02)**	
Augmented aPrEP adjustment (sensitivity analysis)				
Proportion of person-time on PrEP	0.165^e^	0.319^h^	0.195^e^	0.505^h^
Proportion of person-time not on PrEP	0.835^e^	0.681^h^	0.805^e^	0.495^h^
Extrapolated HIV IR^g^ - cases/100 py (95% CI)	3.49 (2.70, 4.27)	2.51 (1.82, 3.20)	5.45 (4.61, 6.29)	2.76 (2.32, 3.20)
HIV infections averted per 100 py^d^ (95% CI)	**0.98** **(0.83**,** 1.13)**		**2.77** **(2.22**,** 3.16)**	

Note: aPrEP = adherent PrEP use

The bold text distinguishes the final estimates of HIV infections averted from the preceding step-by-step calculations

^a^Number of Positive RG Tests/Number of Valid RG Tests. Pooled estimates calculated using imputed data.

^b^Calculated using (Prevalence/Duration = Incidence) formula with Prevalence = RG Estimated Positive Test Rate x 100 and Duration equal to 3 months; RG Positive Test Rate x 100 →RG Positive cases/100 persons over 3 months = RG cases/25 py x 4 → RG cases/100py.

^c^Extrapolated HIV incidence rate calculated using the least square mean estimate and associated CI from the predictive model developed by Mulick and Murray for HIV incidence using RG incidence as a determinant; HIV IR per 100 py = 1.8168 + 0.2218 x (RG IR per 100 py).

^d^Least Square Mean Estimate of (PrEP Adjusted HIV IR at Baseline – PrEP Adjusted HIV IR at Follow-up) with associated CI using the Mulick and Murray model.

^e^Proportion of person time on PrEP calculated assuming 0% person time on PrEP for participants indicating non-adherence at baseline and 100% person time on PrEP for participants indicating adherence at baseline.

^f^Proportion of person time on PrEP calculated assuming 3 months on PrEP for participants indicating adherence to PrEP at both timepoints, 1.5 months on PrEP for participants indicating adherence to PrEP at one timepoint, and 0 months on PrEP for participants indicating non-adherence at both timepoints. Assumes 3 months of observed person-time for each participant.

^g^PrEP Adjusted Extrapolated HIV IR = Least Square Mean Estimate of (Extrapolated HIV IR x Proportion of Person Time not on PrEP) and associated CI using the Mulick and Murray model.

^h^Proportion of person time on PrEP calculated assuming 3 months on PrEP for participants indicating adherence to PrEP at any timepoint and 0 months on PrEP for participants indicating non-adherence at both timepoints. Assumes 3 months of observed person time for each participant.

### CBO Recruitment Approaches

Given the novelty of our CBO delivery strategy for a digital health intervention, we provide additional information on how participants were enrolled into this arm. To make the trial as pragmatic as possible, CBOs were encouraged to propose and develop their own methods of recruitment and retention, building from the suggestions and materials provided in the learning management system. While many CBOs reported where outreach occurred, not all did. CBOs used multiple approaches to recruit and retain participants, including: (1) online recruitment (e.g., on their own website and social media accounts); (2) sexual networking applications (e.g., Grindr); (3) participant referrals; and (4) in-person outreach and community partnerships. Example outreach sites included Pride festivals, university campuses, and local LGBTQ events (e.g., balls, BBQs, and bars). Community partnerships included recruitment at local substance abuse treatment facilities and bathhouses. To retain participants, CBOs primarily used remote communication (i.e., phone calls, text messages, emails, and social media). Almost one-third of CBOs elected to offer space within their organization for participants to complete KIU! in privacy and provided iPads or other devices for participants to use. One CBO also held in-person events for enrolled participants, which included game nights, open discussions, and prize raffles.

Further aligned with the KIU! design as a pragmatic trial, CBOs were able to decide whether to implement participation incentives and, if so, the rates and distribution method. In proposals for funding, CBOs proposed an average of $39 for incentives (range = $0–100) across the duration of KIU!, with 65% allocated to completion of baseline, 6% to completion of main intervention episodes, and 29% to completion of booster episodes. In October 2020, CBOs reported their incentives, with total incentives averaging $40 (range = $0–150): 56% of this amount was allocated to baseline, 16% to main intervention episodes, and 28% to booster episodes. Finally, CBOs reported incentives again near completion of implementation in September 2021. Total incentives at this time averaged $68, with 53% allocated to baseline, 15% to main intervention episodes, and 32% to booster episodes. Some CBOs elected to provide non-financial incentives, including personal lubricant, free sexually transmitted infection (STI) and HIV testing, hand fans, and t-shirts. However, this was not reported uniformly across CBOs. On average, CBOs spent $3560 on incentives. In comparison, DTC participants received an average of $57 in incentives across the intervention. As this trial was a pragmatic design, we aimed to reduce the burden of data reporting. As such, CBOs did not uniformly report incentives in a standardized fashion that would allow for robust analysis of the impact of incentives on implementation.

## Discussion

DHIs can effectively reduce HIV risk and improve HIV care management, but the requirements necessary to scale up effective interventions for widespread reach in real-world settings are not well understood [27, 28]. In this Type III hybrid effectiveness–implementation trial, we reaffirmed the efficacy of KIU! through its effects at increasing aPrEP use [12, 14, 29]. Of particular innovation, this study compared the reach and effectiveness of KIU! across two pragmatic implementation strategies to inform and enhance the future dissemination and implementation of HIV prevention DHIs.

While the DTC arm enrolled a larger number of participants, comprising over 69% of all participants, the CBO arm was able to reach higher proportion of YMSM at elevated HIV risk based on CAS, RG rates, and coming from minoritized communities with high incidence (i.e., Black and Latino). The differences observed between the two arms may be attributable, in part, to study design, as part of the competitive selection process CBOs had to demonstrate a history of recruiting Black and Latino MSM for HIV testing. The DTC staff also had a proven record of recruiting diverse MSM online, but several strategies that were key to past success, such as targeting advertisements to specific populations, were discontinued by popular online social media platforms. While the CBO arm had relative advantages, both arms were successful in recruiting samples that were young, majority racial/ethnic minorities, and at high HIV risk (i.e., based on behavior, STIs, and non-use of PrEP). In other papers, we report on barriers and facilitators of implementing KIU! in CBOs [30] as well as factors that characterize readiness to implement KIU! and other DHIs [31].

Although aPrEP significantly increased in both arms, the CBO arm increased more than DTC. We hypothesize this to reflect the KIU! intervention successfully increasing motivation and skills for aPrEP but that the in-person nature of the CBO arm facilitated direct linkage to PrEP more effectively than the geospatial referral approach used in the DTC arm. CBO participants may also have lived in areas where PrEP was more accessible, thereby facilitating access among those with motivation for use. Indeed, we have previously reported that CBO arm participants reported significantly greater access to sexual health services close to home [32]. CBOs thus may have greater capacity to reach YMSM sub-populations at greater risk for HIV and increase PrEP adherence in these populations. Alternatively, DTC implementation of DHIs may be more effective in mitigating stigma effects, as we have previously reported that it reached more YMSM who had previously avoided HIV testing or talking to providers about sexual health due to perceived stigma [32]. CBOs are useful for individuals willing to visit a space known to provide HIV prevention and/or treatment services, but a DTC strategy can reach those who are unwilling or cannot visit a CBO [33]. Future studies should explore combining the PrEP motivational and behavioral skills benefits of KIU! with telemedicine PrEP delivery to see if it can match the uptake seen in the CBO arm.

Combining study data on reach and effectiveness with micro-costing data allowed us to model costs per infection averted by arm and compare our costing estimates to reported thresholds for cost savings. Our estimated cost per infection averted was more than five times higher in the CBO ($983,029) than the DTC arm ($173,313) in 2021 dollars. To put these estimates in perspective, the CDC cost per HIV infection averted threshold for an intervention to be cost saving was $418,000 in 2012 dollars (inflation adjusted to $547,049 in 2021) [34]. The DTC arm cost 32% of the cost-saving threshold, indicating significant savings to the health system with implementation. Additional background on the micro-costing techniques used and the costs per delivery strategy and per CBO site can be found in another paper [26].

The finding that the more expensive CBO strategy prevents more infections among higher-risk groups is a key aspect of our study. Public health decision-makers often appropriately struggle to balance maximizing intervention reach within a budget and prioritizing higher-risk or disadvantaged groups. Determining whether the extra investment in the CBO strategy provides adequate public health benefit to justify its cost is a question of cost-effectiveness and is beyond the scope of this report of comparative effectiveness of different implementation models for KIU!. We note, however, that the CBO strategy, despite its higher cost, reached a higher proportion Black and Latino YMSM, who generally face higher HIV incidence rates. Other important context is that while the CBO arm reached higher proportions of individuals in key population groups, it reached smaller numbers of such individuals (i.e., DTC arm enrolled 572 Black and Latino YMSM whereas CBO arm enrolled 358). This underscores the importance of targeted interventions that, although potentially more costly, address the specific needs of key populations and contribute significantly to public health by preventing more infections. It is crucial to interpret these results within the broader context of public health and equity, considering the value of prolonging and enriching all lives. In addition, further research outside the context of the COVID-19 pandemic can test strategies to increase efficiencies and lower cost, thereby potentially lowering the cost of CBO implementation. Such work could test strategies for capacity building with CBOs or could use validated approaches to select CBOs that are more ready to implement digital interventions [31].

## Limitations

It is important to contextualize that some of these data were collected during the height of the COVID-19 pandemic when CBOs were closing and setting capacity limitation on HIV testing clinics, and DTC staff were sometimes unable to ship HIV testing kits due to public health restrictions. In our published micro-costing paper, we estimated that costs per participant were 2.72 times higher in this implementation study than in a prior CBO implementation project, suggesting great potential to bring costs down in future implementation below the CDC threshold for cost saving—even in the CBO arm. Furthermore, for nearly all CBOs, KIU! was their first experience delivering a DHI, and efficiencies may be enacted with greater future experience. As such, we anticipate costs will be substantially lower in future implementation.

A considerable amount of STI and follow-up survey data were missing. Having higher levels of missing data is consistent with the pragmatic implementation science design, where some data were reported by CBOs (e.g., baseline STIs), relative to highly controlled effectiveness trials, where all data are collected by researchers. To address this, multiple imputation was used with an imputation model including auxiliary terms shown in preliminary analyses to be associated with the effectiveness outcomes as well as missingness of the effectiveness outcomes. The COVID-19 pandemic also created significant barriers to data collection and required some adaptations to our design (see protocol paper [18]). The length of follow-up was reduced from 12-months to 3-months to allow more time for enrollment and intervention delivery; while this methodological change reduced time to observe sustainment of intervention effectiveness over time, it had the benefit of allowing more time for collecting data relevant to implementation outcomes (i.e., enrollment/reach, fidelity). Since the study was a hybrid type III trial, it was logical to prioritize implementation outcomes over effectiveness outcomes, particularly given that evidence of effectiveness at HIV risk reduction had already been established through 12-months of follow-up with biomedical endpoints [14]. It is a well-known problem that it is a challenge to sustain health behavior changes using DHIs or clinical modalities, so future implementation research on strategies for the delivery of behavior change interventions that can sustain risk reduction is warranted.

At follow-up, HIV testing was not conducted, so to assess the impact of the intervention on averting new HIV infections we utilized a predictive model of HIV incidence [21]. There are limitations of using a predictive model to assess the impact of PrEP on HIV incidence. While these models can provide valuable insights and estimates, they are based on assumptions and may not accurately reflect real-world situations. To mitigate these issues, we conducted a number of sensitivity analyses. Finally, this hybrid trial was specifically targeted toward men between the ages of 18–34 who are attracted to or have sex with men, English-speaking, and not adherent PrEP users prior to the intervention. The effectiveness of KIU! and the ability of CBO and DTC approaches to reach people outside of this population is unknown.

## Conclusions

A previous randomized control trial demonstrated the effectiveness of KIU! at reducing HIV risk—including on biomedical endpoints [14]—earning it a classification by the CDC as “best evidence” [2]. In the current study, we advance understanding of the implementation of this and other effective DHIs through a head-to-head comparison of two viable implementation strategies—DTC and CBO. While existing research highlights the effectiveness of DHIs in increasing uptake and adherence to HIV prevention, to our knowledge this is the first to trial these two delivery strategies. Despite the DTC strategy reaching more YMSM in this study, the CBO strategy was successful in reaching a higher proportion of YMSM at higher risk for HIV. Both delivery strategies show promise and effectiveness in reaching YMSM, enhancing PrEP uptake with adherence, and thereby reducing HIV infections. Future research should address the sustainability of DHIs like KIU! and the requisite systems-level resource and capacity needs for their widespread dissemination and implementation [8]. Future implementation studies may also be able to identify strategies to bring down cost per participant, which is likely viable during delivery outside the height of the COVID-19 pandemic and given the low incremental cost of DHIs. These findings contribute valuable insights to guide and improve the future dissemination and implementation of HIV prevention DHIs, thereby enhancing scalability, reach, and overall public health impact.

More information about KIU!, including implementation support tools, can be found at https://kiu.northwestern.edu/.

## Supplementary Information

Below is the link to the electronic supplementary material.

Supplementary Material 1

Supplementary Material 2

## Data Availability

The datasets used and/or analyzed during the current study are available from the corresponding author on reasonable request.

## Declarations

### Research Involving Human and /or Animal Participants

All procedures performed in studies involving human participants were in accordance with the ethical standards of the institutional and/or national research committee and with the 1964 Helsinki Declaration and its later amendments or comparable ethical standards.
