# Supplementary material for: Comparing Implementation and Effectiveness Outcomes for Two Implementation Strategies of the Keep It Up! Digital HIV Prevention Program: A Type 3 Hybrid Effectiveness-Implementation Trial
Source: AIDS Behav. 2025 Aug 19;29(12):4030–42. doi: 10.1007/s10461-025-04838-0 (PMC12580418; doi:10.1007/s10461-025-04838-0)
Supplement: Supplementary file 1 — Supplementary Material 1 [file 10461_2025_4838_MOESM1_ESM.docx]

AIDS and Behavior

Supplemental Tables

**Comparing Implementation and Effectiveness Outcomes for Two Implementation Strategies of the Keep It Up! Digital HIV Prevention Program: A Type 3 Hybrid Effectiveness-Implementation Trial**

Brian Mustanski, Ph.D., Nanette Benbow, M.A.S., Kathryn Macapagal, Ph.D., Dennis Li, Ph.D., Krystal Madkins, MPH, MSLIS, Rana Saber, M.S., M.S.L., Benjamin Linas, MD, JD Smith, Ph.D., C. Hendricks Brown, Ph.D., Sarah Munroe, MPH, Susheel Reddy, MPH, Bruce R. Schackman, Ph.D., Gregory Swann, M.S., M.A., Patrick Janulis, Ph.D., alithia zamantakis, Ph.D., Juan Pablo Zapata, Ph.D.

Corresponding Author: Brian Mustanski, Ph.D., Institute for Sexual and Gender Minority Health and Wellbeing, Northwestern University, 625 N. Michigan Ave. Floor 14, Chicago, IL 60611, United States; Email: [brian@northwestern.edu](mailto:brian@northwestern.edu)

**Table S1** Loss to Follow-Up by Recruitment Strategy, Outcomes, Demographic and Auxiliary Variables: 2020-2023

|  | **Completed Follow-up**  **(n = 1413)** | **Lost to Follow-up**  **(n = 711)** | **P-value** | **Total**  **(n = 2124)** |
| --- | --- | --- | --- | --- |
|  |  |  |  |  |
| **Study Arm, n (%)** |  |  | 0.0006^a^ |  |
| CBO | 402 (28.5) | 254 (35.7) |  | 656 (30.9) |
| DTC | 1011 (71.6) | 457 (64.3) |  | 1468 (69.1) |
|  |  |  |  |  |
| **Age at Baseline (years), n (%)** |  |  | < 0.0001^b^ |  |
| 18 – 21 | 239 (16.9) | 202 (28.4) |  | 441 (20.8) |
| 22 – 25 | 390 (27.6) | 226 (31.8) |  | 616 (29.0) |
| 26 - 29 | 437 (30.0) | 170 (23.9) |  | 607 (28.6) |
| > 30 | 347 (24.6) | 113 (15.9) |  | 460 (21.7) |
|  |  |  |  |  |
| **Gender at Baseline, n (%)** |  |  | 0.2989^a^ |  |
| Man | 1319 (93.4) | 655 (92.1) |  | 1974 (92.9) |
| Gender nonconforming | 94 (6.7) | 56 (7.9) |  | 150 (7.1) |
|  |  |  |  |  |
| **Sexual Orientation at Baseline, n (%)** |  |  | 0.0248^a^ |  |
| Gay | 1060 (75.0) | 501 (70.5) |  | 1561 (73.5) |
| Other | 353 (25.0) | 210 (29.5) |  | 563 (26.5) |
|  |  |  |  |  |
| **Race/Ethnicity at Baseline, n (%)** |  |  | < 0.0001^a^ |  |
| White | 583 (41.3) | 247 (34.7) |  | 830 (39.1) |
| Black or African-American | 170 (12.0) | 143 (20.1) |  | 313 (14.7) |
| Latinx | 408 (28.9) | 209 (29.4) |  | 617 (29.1) |
| Other | 252 (17.8) | 112 (15.8) |  | 364 (17.1) |
|  |  |  |  |  |
| **aPrEP Last 30 Days – Baseline, n (%)** |  |  | 0.0001^a^ |  |
| Yes | 278 (19.7) | 92 (12.9) |  | 370 (17.4) |
| No | 1135 (80.3) | 619 (87.1) |  | 1754 (82.6) |
| Missing^c^ | 0 | 0 |  | 0 |
|  |  |  |  |  |
| **PrEP Use – Baseline, n (%)** |  |  | 0.0003^a^ |  |
| Yes | 329 (23.3) | 117 (16.5) |  | 446 (21.0) |
| No | 1084 (76.7) | 594 (83.5) |  | 1678 (79.0) |
| Missing^c^ | 0 | 0 |  | 0 |
|  |  |  |  |  |
| **Rectal Gonorrhea Lab Result – Baseline, n (%)** |  |  | 0.3126^d^ |  |
| Positive | 27 (1.9) | 7 (1.0) |  | 34 (1.6) |
| Negative | 781 (55.3) | 127 (17.9) |  | 908 (42.8) |
| Missing, Not done, or Invalid^c^ | 605 (42.8) | 577 (81.2) |  | 1182 (55.7) |
|  |  |  |  |  |
| **Urethral Gonorrhea Lab Result – Baseline, n (%)** |  |  | 0.3481^d^ |  |
| Positive | 15 (1.1) | 5 (0.7) |  | 20 (0.9) |
| Negative | 844 (59.7) | 158 (22.2) |  | 1002 (47.2) |
| Missing, Not done, or Invalid^c^ | 554 (39.2) | 548 (77.1) |  | 1102 (51.9) |
|  |  |  |  |  |
| **Rectal Chlamydia Lab Result – Baseline, n (%)** |  |  | 0.1107^a^ |  |
| Positive | 44 (3.1) | 12 (1.7) |  | 56 (2.6) |
| Negative | 765 (54.1) | 122 (17.2) |  | 887 (41.8) |
| Missing, Not done, or Invalid^c^ | 604 (42.8) | 577 (81.2) |  | 1181 (55.6) |
|  |  |  |  |  |
| **Uerthral Chlamydia Lab Result – Baseline, n (%)** |  |  | 0.4307^d^ |  |
| Positive | 22 (1.6) | 6 (0.8) |  | 28 (1.3) |
| Negative | 837 (59.2) | 157 (22.1) |  | 994 (46.8) |
| Missing, Not done, or Invalid^c^ | 554 (39.2) | 548 (77.1) |  | 1102 (51.9) |
|  |  |  |  |  |
| **Number of Cisgender Male Sex Partners in Past 3 Months, No Condom – Baseline, n (%)** |  |  | 0.9735^e^ |  |
| N | 1413 | 711 |  | 2124 |
| Mean (SD) | 2.2 (4.09) | 2.3 (4.72) |  | 2.2 (4.31) |
| Median | 1.0 | 1.0 |  | 1.0 |
| IQR | 0 – 3.0 | 0 – 3.0 |  | 0 – 3.0 |
| Minimum, Maximum | 0, 70 | 0, 80 |  | 0, 80 |
|  |  |  |  |  |
| **Number of Anal/Vaginal Sex Partners in Past 3 Months – Baseline, n (%)** |  |  | 0.3108^e^ |  |
| N | 1413 | 711 |  | 2124 |
| Mean (SD) | 3.3 (5.07) | 3.6 (7.09) |  | 3.4 (5.83) |
| Median | 2.0 | 2.0 |  | 2.0 |
| IQR | 1.0 – 4.0 | 1.0 – 4.0 |  | 1.0 – 4.0 |
| Minimum, Maximum | 0, 70 | 0, 100 |  | 0, 100 |
|  |  |  |  |  |
| Note: aPrEP = adherent PrEP use  ^a^P-value calculated using a chi-square test.  ^b^ P-value calculated using the chi-square correlation statistic.  ^c^Not considered when calculating p-value.  ^d^P-value calculated using a Fisher’s exact test.  ^e^P-value calculated using a two-sample t-test assuming unequal variances using Satterthwaite degrees of freedom. | | | | |

**Table S2** Auxiliary Variables Used in Imputation Model by Recruitment Strategy: 2020-2023

|  | **DTC**  **(n = 1468)** | **CBO**  **(n = 656)** | **P-value** | **Total**  **(n = 2124)** |
| --- | --- | --- | --- | --- |
|  |  |  |  |  |
| **PrEP Use – Baseline, n (%)** |  |  | 0.0073^a^ |  |
| Yes | 285 (19.4) | 161 (24.5) |  | 446 (21.0) |
| No | 1183 (80.6) | 495 (75.5) |  | 1678 (79.0) |
| Missing^b^ | 0 | 0 |  | 0 |
|  |  |  |  |  |
| **PrEP Use – Follow-up, n (%)** |  |  | < 0.0001^a^ |  |
| Yes | 295 (20.1) | 112 (17.1) |  | 407 (19.2) |
| No | 671 (45.7) | 130 (19.8) |  | 801 (37.7) |
| Missing^b^ | 502 (34.2) | 414 (45.2) |  | 916 (43.1) |
|  |  |  |  |  |
| **Urethral Gonorrhea Lab Result – Baseline, n (%)** |  |  | < 0.0001^a^ |  |
| Positive | 2 (0.1) | 18 (2.7) |  | 20 (0.9) |
| Negative | 637 (43.4) | 365 (55.6) |  | 1002 (47.2) |
| Missing, Not done, or Invalid^b^ | 829 (56.5) | 273 (41.6) |  | 1102 (51.9) |
|  |  |  |  |  |
| **Urethral Gonorrhea Lab Result – Follow-up, n (%)** |  |  | 0.0004^a^ |  |
| Positive | 1 (0.1) | 6 (0.9) |  | 7 (0.3) |
| Negative | 711 (48.4) | 180 (27.4) |  | 891 (42.0) |
| Missing, Not done, or Invalid^b^ | 756 (51.5) | 470 (71.7) |  | 1226 (57.7) |
|  |  |  |  |  |
| **Rectal Chlamydia Lab Result – Baseline, n (%)** |  |  | 0.0034^a^ |  |
| Positive | 28 (1.8) | 28 (4.3) |  | 56 (2.6) |
| Negative | 611 (41.6) | 276 (42.1) |  | 887 (41.8) |
| Missing, Not done, or Invalid^b^ | 829 (56.5) | 352 (53.7) |  | 1181 (55.6) |
|  |  |  |  |  |
| **Rectal Chlamydia Lab Result – Follow-up, n (%)** |  |  | 0.0255^a^ |  |
| Positive | 27 (1.8) | 13 (2.0) |  | 40 (1.9) |
| Negative | 682 (46.5) | 153 (23.3) |  | 835 (39.3) |
| Missing, Not done, or Invalid^b^ | 759 (51.7) | 490 (74.7) |  | 1249 (58.8) |
|  |  |  |  |  |
| **Urethral Chlamydia Lab Result – Baseline, n (%)** |  |  | 0.0293^a^ |  |
| Positive | 12 (0.8) | 16 (2.4) |  | 28 (1.3) |
| Negative | 627 (42.7) | 367 (56.0) |  | 994 (46.8) |
| Missing, Not done, or Invalid^b^ | 829 (56.5) | 273 (41.6) |  | 1102 (51.9) |
|  |  |  |  |  |
| **Urethral Chlamydia Lab Result – Follow-up, n (%)** |  |  | 0.0208^a^ |  |
| Positive | 8 (0.5) | 7 (1.1) |  | 15 (0.7) |
| Negative | 704 (48.0) | 179 (27.3) |  | 883 (41.6) |
| Missing, Not done, or Invalid^b^ | 756 (51.5) | 470 (71.7) |  | 1226 (57.7) |
|  |  |  |  |  |
| **Number of Anal/Vaginal Sex Partners in Past 3 Months – Baseline, n (%)** |  |  | 0.8044^c^ |  |
| n | 1468 | 656 |  | 2124 |
| Mean (SD) | 3.4 (6.13) | 3.5 (5.09) |  | 3.4 (5.83) |
| Median | 2.0 | 2.0 |  | 2.0 |
| IQR | 1.0 - 4.0 | 1.0 - 4.0 |  | 1.0 - 4.0 |
| Minimum, Maximum | 0, 100 | 0, 80 |  | 0, 100 |
|  |  |  |  |  |
| **Number of Anal/Vaginal Sex Partners in Past 3 Months – Follow-up, n (%)** |  |  | 0.7357^c^ |  |
| n | 968 | 242 |  | 1210 |
| Mean (SD) | 2.7 (4.65) | 2.8 (4.37) |  | 2.7 (4.59) |
| Median | 1.0 | 1.0 |  | 1.0 |
| IQR | 1.0 - 3.0 | 1.0 - 3.0 |  | 1.0 - 3.0 |
| Minimum, Maximum | 0, 85 | 0, 45 |  | 0, 85 |
|  |  |  |  |  |
| ^a^P-value calculated using a chi-square test.  ^b^Not considered when calculating p-value.  ^c^ P-value calculated using a two-sample t-test assuming unequal variance using Satterthwaite degrees of freedom. | | | | |

**Table S3** Pooled Estimates of Bivariate Results by Recruitment Strategy Using Imputed Data^a^: 2020-2023

|  | **DTC**  **(n = 1468)** | **CBO**  **(n = 656)** | **P-value** | **Total**  **(n = 2124)** |
| --- | --- | --- | --- | --- |
|  |  |  |  |  |
| **Rectal Gonorrhea Lab Result – Baseline, (%)** |  |  | 0.0003^b^ |  |
| Positive | 2.7 | 5.6 |  | 3.6 |
| Negative | 97.3 | 94.4 |  | 96.4 |
|  |  |  |  |  |
| **Rectal Gonorrhea Lab Result – Follow-up, (%)** |  |  | 0.0745^b^ |  |
| Positive | 2.1 | 4.2 |  | 2.8 |
| Negative | 97.9 | 95.8 |  | 97.3 |
|  |  |  |  |  |
| **PrEP Use – Baseline** | No missing data. See Table S2. | | | |
|  |  |  |  |  |
| **PrEP Use – Follow-up, (%)** |  |  | 0.0023^b^ |  |
| Yes | 29.6 | 45.9 |  | 34.6 |
| No | 70.4 | 54.1 |  | 65.4 |
|  |  |  |  |  |
| **Number of Cisgender Male Sex Partners in Past 3 Months, No Condom – Baseline** | No missing data. See Table 1. | | | |
|  |  |  |  |  |
| **Number of Cisgender Male Sex Partners in Past 3 Months, No Condom – Follow-up** |  |  | 0.0247^c^ |  |
| Mean (SE) | 2.1 (0.14) | 3.0 (0.39) |  | 2.4 (0.17) |
|  |  |  |  |  |
| **aPrEP Last 30 Days – Baseline** | No missing data. See Table 1. | | | |
|  |  |  |  |  |
| **aPrEP Last 30 Days – Follow-up, (%)** |  |  | 0.0021^b^ |  |
| Yes | 27.6 | 44.2 |  | 32.7 |
| No | 72.4 | 55.8 |  | 67.3 |
|  |  |  |  |  |
| **Urethral Gonorrhea Lab Result – Baseline, (%)** |  |  | 0.0002^b^ |  |
| Positive | 1.6 | 4.8 |  | 2.6 |
| Negative | 98.4 | 95.2 |  | 97.4 |
|  |  |  |  |  |
| **Urethral Gonorrhea Lab Result – Follow-up, (%)** |  |  | 0.0793^b^ |  |
| Positive | 1.3 | 3.2 |  | 1.9 |
| Negative | 98.7 | 96.8 |  | 98.1 |
|  |  |  |  |  |
| **Rectal Chlamydia Lab Result – Baseline, (%)** |  |  | 0.1105^b^ |  |
| Positive | 6.1 | 9.0 |  | 7.0 |
| Negative | 93.9 | 91.0 |  | 93.0 |
|  |  |  |  |  |
| **Rectal Chlamydia Lab Result – Follow-up, (%)** |  |  | 0.1858^b^ |  |
| Positive | 5.5 | 9.0 |  | 6.6 |
| Negative | 94.5 | 91.0 |  | 93.4 |
|  |  |  |  |  |
| **Urethral Chlamydia Lab Result – Baseline, (%)** |  |  | 0.1068^b^ |  |
| Positive | 4.7 | 6.8 |  | 5.3 |
| Negative | 95.3 | 93.2 |  | 94.7 |
|  |  |  |  |  |
| **Urethral Chlamydia Lab Result – Follow-up, (%)** |  |  | 0.1392^b^ |  |
| Positive | 3.6 | 7.0 |  | 4.6 |
| Negative | 96.4 | 93.0 |  | 95.4 |
|  |  |  |  |  |
| **Number of Anal/Vaginal Sex Partners in Past 3 Months – Baseline** | No missing data. See Table S2. | | | |
|  |  |  |  |  |
| **Number of Anal/Vaginal Sex Partners in Past 3 Months – Follow-up** |  |  | 0.0490^c^ |  |
| Mean (SE) | 3.0 (0.17) | 3.9 (0.44) |  | 3.3 (0.19) |
|  |  |  |  |  |
| Note: aPrEP = adherent PrEP use  ^a^Percentages, mean estimates, and standard errors shown are estimates pooled across imputed datasets.  ^b^P-value calculated using pooled estimate of chi-square statistic while applying the Wilson-Hiferty transformation.  ^c^P-value calculated using pooled estimate of two sample t-test using Satterthwaite degrees of freedom. Unequal variances are assumed. | | | | |
