# Supplementary material for: Comparing Implementation and Effectiveness Outcomes for Two Implementation Strategies of the Keep It Up! Digital HIV Prevention Program: A Type 3 Hybrid Effectiveness-Implementation Trial
Source: AIDS Behav. 2025 Aug 19;29(12):4030–42. doi: 10.1007/s10461-025-04838-0 (PMC12580418; doi:10.1007/s10461-025-04838-0)
Supplement: Supplementary file 2 — Supplementary Material 2 [file 10461_2025_4838_MOESM2_ESM.docx]

AIDS and Behavior

Supplemental Methods

**Comparing Implementation and Effectiveness Outcomes for Two Implementation Strategies of the Keep It Up! Digital HIV Prevention Program: A Type 3 Hybrid Effectiveness-Implementation Trial**

Brian Mustanski, Ph.D., Nanette Benbow, M.A.S., Kathryn Macapagal, Ph.D., Dennis Li, Ph.D., Krystal Madkins, MPH, MSLIS, Rana Saber, M.S., M.S.L., Benjamin Linas, MD, JD Smith, Ph.D., C. Hendricks Brown, Ph.D., Sarah Munroe, MPH, Susheel Reddy, MPH, Bruce R. Schackman, Ph.D., Gregory Swann, M.S., M.A., Patrick Janulis, Ph.D., alithia zamantakis, Ph.D., Juan Pablo Zapata, Ph.D.

Corresponding Author: Brian Mustanski, Ph.D., Impact Institute, Northwestern University, 625 N. Michigan Ave. Floor 14, Chicago, IL 60611, United States; Email: [brian@northwestern.edu](mailto:brian@northwestern.edu)

**Supplemental Methods**

**Data Collection**

Sexually transmitted infections (STIs) data collection differed between the two arms. Using detailed multimedia instructions, participants in the DTC condition self-collected a urine sample and rectal swab at baseline and 12-week follow-up using the Aptima Urine and Multisite Swab Specimen Collection Kits, respectively. Testing for Gonorrhea and Chlamydia (GC/CT) was performed with the Aptima Combo 2 GC/CT nucleic acid amplification test. Positive cases were provided referrals to local clinics that offer free or low-cost treatment and were reported to the responsible health department according to requirements. CBO-arm participants were tested for rectal and urethral gonorrhea and chlamydia at their CBO at baseline and 12-week follow-up. CBO participants were asked for electronic consent for researchers to obtain their STI results from the CBO. For those who consented, CBOs transferred their results to the research team via a secure web portal. In instances where the CBO did not do STI testing, the participant did not want to return to the CBO for follow-up testing, or the CBO was unable to bring the participant back in for testing, CBO participants who agreed to be shipped an STI test kit were tested using the same methods as their DTC counterparts.

**Measures**

*Self-Report*

Daily PrEP users were asked “In the last MONTH (30 days), has there been a time when you did NOT take PrEP for 4 or more days in a row?” and PrEP users who reported taking PrEP on demand were asked “You indicated using PrEP “on demand” or with a “2-1-1” dosing schedule. In the last MONTH (30 days), has there been a time when you MISSED any doses of PrEP before or after having sex?” Other measures relevant to behavioral risk and study disposition were used as auxiliary variables needed for the imputation of missing outcome and PrEP adherence data. The number of self-reported anal or vaginal sex partners (“In the PAST 3 MONTHS, with how many partners have you had anal or vaginal sex?”) was collected at baseline and follow-up surveys. PrEP use data was collected using a survey item addressing current PrEP use (“Are you CURRENTLY taking PrEP to reduce the risk of getting HIV?”) with “yes” and “no” response options.

*Sexually Transmitted Infections*

Urethral gonorrhea, rectal chlamydia, and urethral chlamydia test results were obtained from swabs or urine samples collected at both timepoints.

*Metadata*

The number of KIU! study modules completed was recorded by the software platform, with completion of seven modules indicating completion of the main KIU! intervention and completion of nine modules indicating completion of the main intervention plus the two additional booster sessions. The number of modules completed was characterized as a continuous variable.

**PrEP Adherence**

Proportion of person-time on PrEP was estimated using the PrEP adherence measures collected at baseline and follow-up. The proportion of person-time on PrEP was calculated as the quotient of attributed person-time on PrEP and total observed person-time. For each of the study-arm-specific follow-up estimates, those indicating adherence at both baseline and follow-up were assumed to present 3 months on PrEP; those indicating adherence at one timepoint and non-adherence at the other were assumed to present 1.5 months on PrEP; those indicating non-adherence at both timepoints were assumed to present 0 months on PrEP. Accumulated person-time on PrEP across participants for a given study arm was summed and divided by total person-time observed with each subject contributing 3 months observed person-time. For the arm-specific baseline estimates, participants indicating 30-day adherence at baseline were assumed to present 100% person-time on PrEP; those indicating non-adherence at baseline were assumed to present 0% person-time on PrEP.

***Demographics***

An additional 114 participants who consented and met nearly all enrollment criteria, except for completion of the baseline survey, were incorporated into the multiple imputation model to improve estimates of missingness but were excluded from all outcome analyses.

***HIV Infections Averted***

To model the incidence of HIV infection per 100 person-years, in each arm, we estimated the incidence of RG based on study results then imputed the likely rate of HIV infection based on the incidence of RG infection, using previous estimates from the literature that related RG and HIV incidence among men who have sex with men (Mullick & Murray, 2020). Finally, we adjusted the estimated incidence of HIV infection to reflect PrEP use in each arm because HIV PrEP disrupts the correlation between incidence of HIV and other STIs due to its protective effects for only HIV.

**Step 1.** **Estimate incidence of RG in Keep It Up! study**. We calculated the numerator of the incidence of RG as the number of positive RG tests (using all test results including those that were imputed). To estimate the denominator (i.e., person-time exposed), we assumed that RG infections were randomly distributed during follow-up, such that on average, infections occurred at the mid-point of follow-up.

**Step 2.** **Translate incidence of RG into unadjusted incidence of HIV**. Mullick and Murray previously published a multi-variable controlled model relating empirically observed RG incidence to the incidence of HIV infection that would be observed were it possible to enroll a large enough sample (Mullick & Murray, 2020). We used that regression equation to estimate the unadjusted HIV incidence in both arms of the study. We used the 5^th^ and 95^th^ percentiles of the confidence intervals from that equation to provide an estimate of uncertainty from projections. Importantly, these intervals are relevant to the variability in the quantified relationship between RG and HIV incidence rates (IRs) as defined by the Mulick and Murray regression model but do not incorporate uncertainty in the effect sizes ascribed to study arm or timepoint.

**Step 3**. **Adjust HIV incidence for the effect of PrEP.** Because PrEP has no effect on RG incidence but does prevent HIV transmission, PrEP adherence tends to appropriately bias the Mulick and Murray method by disassociating RG from HIV. To approximate the effect of PrEP on regression-predicted HIV IRs, we multiplied the estimated HIV incidence by the proportion of person-time not taking PrEP.

***Missingness***

Differences in follow-up status by study arm as well as demographic and outcome measures are shown in Table S1. Participants in the DTC condition were more likely to complete follow-up compared to CBO participants (p = 0.0006). Older participants were more likely to complete follow-up compared to younger participants (p < .0001). Participants who completed follow-up were more likely to be White and less likely to be Black compared to participants who did not complete follow-up (p < .0001). Gay participants completed follow-up at a higher rate as compared to participants reporting another sexual orientation (p = 0.0248). There were no significant differences in follow-up completion by gender identity.

Participants who contributed to follow-up were more likely to report using PrEP (p = 0.0003) and report as an adherent PrEP user at baseline (p = 0.0001). There were no significant differences on follow-up completion based on rectal or urethral gonorrhea or chlamydia. There were also no significant differences based on number of anal/vaginal sex partners or condomless cisgender male sex partners. All variables related to missingness were included in the imputation model as either primary or auxiliary variables.
